# Hypidone Hydrochloride (YL-0919), a Sigma-1 Receptor Agonist, Improves Attention by Increasing BDNF in mPFC

**DOI:** 10.3390/ph18040455

**Published:** 2025-03-24

**Authors:** Yixin Yang, Yue Zhang, Xiaojuan Hou, Hailong Li, Hui Ma, Yunfeng Li

**Affiliations:** 1 Beijing Institute of Basic Medical Sciences, Beijing 100000, China; yyx9990223@163.com; 2 Department of Anesthesiology, Capital Medical University, Beijing 100000, China; zhangyue971019@163.com; 3 Department of postgraduate, Hebei North University, Zhangjiakou 075000, China; houxiaojuan0509@163.com; 4 Department of Clinical Pharmacy, Guangdong Pharmaceutical University, Guangzhou 510000, China; 13060673854@163.com; 5 Beijing Key Laboratory of Neuropsychopharmacology, State Key Laboratory of Toxicology and Medical Countermeasures, Beijing Institute of Pharmacology and Toxicology, Beijing 100000, China

**Keywords:** attention deficit, YL-0919, mPFC, sigma-1 receptor, BDNF

## Abstract

**Background/Objectives:** The available treatment for attention deficit is drug therapy, but the drugs show poor adverse effect profiles and individual variability in response, especially in adults. Hypidone hydrochloride (YL-0919) is a selective sigma-1 receptor agonist that demonstrated a faster onset antidepressant effect in our previous studies. Current studies aim to study the attention-enhancing effect and mechanism of YL-0919. **Methods:** We used the five-choice serial reaction time task (5-CSRTT) to measure the attention-improving effect of YL-0919 in SD rats under a physiological state and exogenous corticosterone (CORT)-exposed state. The depression/anxiety-like behavioral experiments were used in the CORT-exposed rats. Immunofluorescence staining, western blotting, and Golgi–Cox staining were used to investigate the attention-improving mechanism of YL-0919. **Results:** The studies found that intragastric administration of 2.5 and 5 mg/kg YL-0919 for 6 days significantly improved the attention of SD rats under a physiological state. CORT exposure caused depression/anxiety-like behaviors and attention deficit in the rats. Intragastric administration of 3 mg/kg SA4503 or 2.5 and 5 mg/kg YL-0919 for 6 days significantly alleviated attention deficit in SD rats under an exogenous CORT-exposed state. In addition, YL-0919 administration obviously increased the expression of BDNF, PSD95, and synapsin1 and improved the dendritic complexity and the dendritic spine density in the medial prefrontal cortex (mPFC). **Conclusions:** These results reveal that YL-0919 as a selective sigma-1 receptor agonist can significantly improve the attention of SD rats under a physiological state and exogenous CORT-exposed state. Improving the level of BDNF and dendritic complexity in the mPFC may be the important mechanisms of YL-0919 to improve attention. The study also provides a potential novel target for the drug therapy of attention deficit.

## 1. Introduction

Attention deficit is a common clinical manifestation in many neuropsychiatric diseases, such as attention deficit hyperactivity disorder [1], generalized anxiety disorder, and depressive disorder [2]. The available treatment for attention deficit is drug therapy, but the drugs show poor adverse effect profiles [3] and individual variability in response, especially in adults [4]. Recent research on disorders that cause attention deficit and treatments are obviously insufficient. Therefore, it is necessary to develop new potential targets and mechanisms for attention deficit associated with neuropsychiatric diseases.

The sigma-1 receptor, a chaperone protein located in the cholesterol-rich region of the endoplasmic reticulum (ER) mitochondria-associated membrane (MAM) [5], plays an important role in the protection of neurons, such as synaptogenesis and myelination in the brain, through various signal pathways [6]. The sigma-1 receptor is expressed in neurons, cerebral microglia, and astrocytes [7]. Its function is associated with the development of many neurological and psychiatric disorders, including addiction, schizophrenia, depression, and Parkinson’s disease [8]. It has been shown that treatment with SA-4503, which is currently recognized as a sigma-1 receptor agonist, enhances synaptic abnormalities to ameliorate cognitive dysfunction [9]. Therefore, the sigma-1 receptor can be a potential target for the drug therapy of emotional and cognitive disorders [10]. At present, although attention is the basis of self-regulated cognition [11], there are few studies on whether the sigma-1 receptor can improve attention behaviors.

The literature suggests that some drugs can be used for the treatment of depression and attention deficit [12]. Hypidone hydrochloride (YL-0919), a selective sigma-1 receptor agonist [13], was developed as an antidepressant candidate in our laboratory and is currently in phase II clinical trials. We verified that YL-0919 had a faster onset (3–9 d) antidepressant effect [13,14,15]. Previous studies in our laboratory have found that YL-0919 has the characteristics of a selective sigma-1 receptor agonist [13]. The research showed that YL-0919 could activate the BDNF-mTOR pathway to enhance synaptic plasticity in the prefrontal cortex (PFC) and hippocampus [16,17]. This study aims to research the attention-improving effect and mechanism of YL-0919 to explore whether the sigma-1 receptor could be a therapeutic target for attention deficit.

PFC plays a role in the modulation of attention levels. Research revealed that fast-spiking parvalbumin neurons in the medial prefrontal cortex (mPFC) showed increasing and continuous firing because of attention driven by goals, which was correlated with a high attention level [18]. Researchers speculate that the damage to this key area may lead to attention deficits, with the possibility of the simultaneous occurrence of cognitive and emotional symptoms [19]. Therefore, researchers believe that the mPFC may act as a cognitive/emotional integration site [20]. In addition, researchers have shown that mPFC controls the activity of the sensory thalamus via a pathway within the basal ganglia [21]. The pathway can activate the mPFC to regulate which sensory information is prioritized and which is suppressed, thereby inhibiting distraction. In other words, the subcortical structures also have an essential function in attention.

Although the accurate etiology of attention deficit has not been confirmed yet, several lines of evidence from clinical research [22,23] suggested that neurotrophins (NTs), including BDNF, take part in the pathogenesis and mechanism of biological treatments of attention deficit [24]. The literature suggests that after ingesting CORT at 40 mg/kg/d for 21 days, rats are under a depression/anxiety-like state [25]. Moreover, the study revealed that after exposure to exogenous CORT, the sigma-1 receptor participated in the behavioral influence of ketamine. It also contributed to the restoration of the structure and function in the mPFC^Pyr^ neuron as the level of BDNF increased [26]. We speculate that serving as a selective sigma-1 receptor agonist, YL-0919 may have the faster onset attention-enhancing effects by improving neuroplasticity with increasing protein in the mPFC, including BDNF.

To directly test our hypothesis, we used the five-choice serial reaction time task (5-CSRTT) [27] to measure the attention-improving effect of YL-0919 in SD rats under a physiological state and exogenous CORT-exposed state. In this study, we tested SA-4503 with two different doses of YL-0919 to explore whether the sigma-1 receptor has a universal effect on improving attention. We also used immunofluorescence staining and western blotting to test the expression of BDNF, PSD95, and synapsin1 and Golgi–Cox staining to investigate the dendritic complexity of neurons in the mPFC. We hope that the mechanism of the effect of YL-0919 on improving attention was explored to provide new drug treatment ideas for increasing attention and relieving attention deficit.

## 2. Results

### 2.1. YL-0919 Improves the Attention of Rats Under a Physiological State

**Figure 1:**
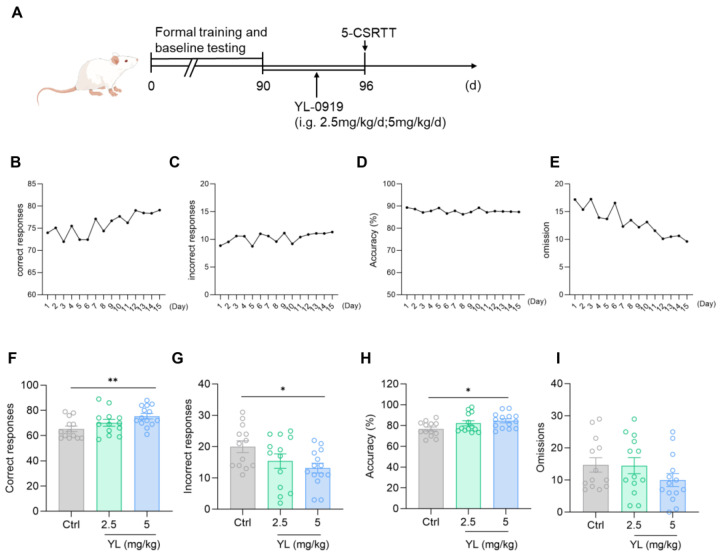
YL-0919 increases attention in rats under a physiological state. (**A**) Design of the behavioral experiment. “(d)” represents the number of days. (**B**–**E**) According to the requirements of the 5-CSRTT experiment, four indicators reflecting the attention of SD rats were used to obtain the unified baseline. A portion of the training data was selected for the illustration. (**F**) Number of correct responses for the 5-CSRTT. (**G**) Number of incorrect responses for the 5-CSRTT. (**H**) Accuracy rate of the 5-CSRTT. (**I**) Numbers of omissions for the 5-CSRTT. Data analysis used one-way ANOVA. Dunnett’s multiple comparisons test was performed for comparisons among groups. The data are presented in the form of mean values. * *p* < 0.05 and ** *p* < 0.01, n = 13.

After training was complete and a unified baseline was obtained (Figure 1B–E), the experimental animals under a physiological state were gavaged with 2.5 mg/kg or 5 mg/kg YL-0919 for 6 days (Figure 1A). The results revealed that compared with the rats in the control group, the attention of the rats in the administration group was significantly increased (*F*_(2,37)_ = 4.699, *p* = 0.0152, Figure 1F; *F*_(2,37)_ = 3.306, *p* = 0.0478, Figure 1G; *F*_(2,37)_ = 3.666, *p* = 0.0353, Figure 1H) based on the 5-CSRTT. Compared with those in the control group, the number of correct responses in the 2.5 mg/kg YL-0919 group did not increase (*p* = 0.2670, Figure 1F), and the number of incorrect responses did not decrease (*p* = 0.1780, Figure 1G). The number of correct responses increased in the 5 mg/kg YL-0919 group (*p* = 0.0078, Figure 1F), the number of incorrect responses decreased (*p* = 0.0292, Figure 1G), and the accuracy rate increased (*p* = 0.0216, Figure 1H), suggesting that YL-0919 (5 mg/kg) can increase the attention of animals under a physiological state.

### 2.2. YL-0919 Improves Attention by Increasing Neuronal Excitability and the Expression of BDNF and Synaptic-Related Proteins Under a Physiological State

**Figure 2:**
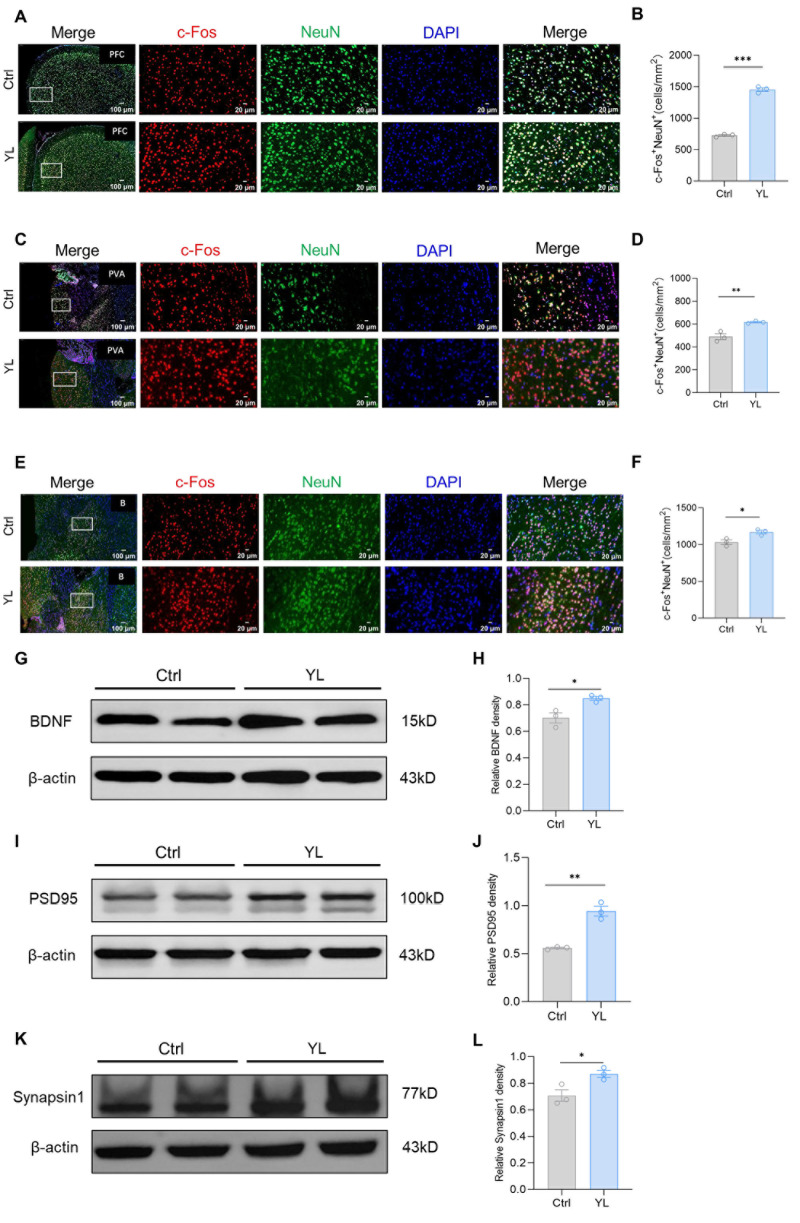
YL-0919 stimulates attention-related brain regions and increases the expression of BDNF, PSD95, and synapsin1. (**A**) Representative images of NeuN (green) and c-Fos (red) immunostaining in the mPFC of the control group and YL group. (**B**) Quantification of NeuN^+^ and c-Fos^+^ double-positive cells in the mPFC. (**C**) Representative images of NeuN (green) and c-Fos (red) immunostaining in the paraventricular nucleus of the hippocampus (PVA) in the control and YL groups. (**D**) Quantification of NeuN^+^ and c-Fos^+^ double-positive cells in the PVA. (**E**) Representative images of NeuN (green) and c-Fos (red) immunostaining in the basal ganglia of the control and YL groups. (**F**) Quantification of NeuN^+^ and c-Fos^+^ double-positive cells in the basal ganglia. (**G**) Western blotting results showing the BDNF levels in the control and YL groups. (**H**) Relative grayscale value of BDNF. (**I**) Western blotting results showing the PSD95 levels in the control and YL groups. (**J**) Relative grayscale value of PSD95. (**K**) Western blotting results showing the synapsin1 levels in the control and YL groups. (**L**) Relative grayscale value of synapsin1. In the western blotting, the spliced fragments originate from the same original image. Data analysis using a *t* test revealed that * *p* < 0.05, ** *p* < 0.01, and *** *p* < 0.001; the data are presented in the form of mean values, n = 3.

Previous studies have shown that brain regions such as the mPFC, basal ganglia, and thalamus may be involved in the regulation of attention. We used immunofluorescence staining to label c-FOS and NeuN in relevant brain regions of the control group and the drug administration group. The number of c-FOS and NeuN double-positive cells in the group treated with 5 mg/kg YL-0919 increased compared to the values in the control group in the relevant regions, indicating that YL-0919 improved attention by stimulating mature neurons in certain brain regions. The results revealed that, compared to those in the control group, the number of c-FOS and NeuN double-positive cells in the mPFC (*p* < 0.0001, Figure 2A,B), anterior paraventricular nucleus of the thalamus (*p* = 0.0011, Figure 2C,D), and basal ganglia (*p* = 0.0255, Figure 2E,F) of the rats that received 5 mg/kg YL-0919 was significantly higher. In particular, the increase in the number of mature neurons in the mPFC was more significant (Figure 2B). Moreover, we used western blotting to measure the expression of BDNF (*p*_(H)_ = 0.0217, Figure 2G,H), PSD95 (*p*_(J)_ = 0.0017, Figure 2I,J), and synapsin1 (*p*_(L)_ = 0.033, Figure 2K,L) in the PFC. As shown in the figure, compared with those in the control group, the levels of three proteins were significantly increased in the PFC of the rats treated with 5 mg/kg YL-0919. These findings suggest that YL-0919 can improve attention by stimulating neuronal excitability and improving the expression of BDNF, PSD95, and synapsin1 in the mPFC.

### 2.3. Exposure to Exogenous CORT Results in Anxiety/Depression-like Behaviors and Attention Deficit and YL-0919 Significantly Improves the Attention of Rats Under This State

**Figure 3:**
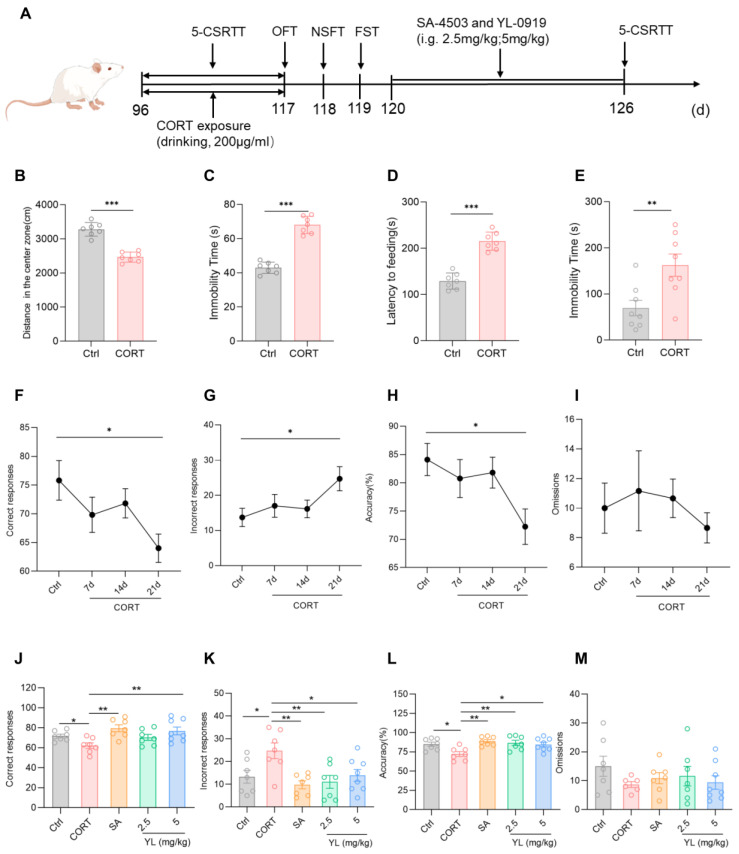
YL-0919 significantly improved attention in rats under a CORT-exposed state. (**A**) Design of the behavioral experiment. “(d)” represents the number of days. (**B**) Movement distance in the central region of the field in the OFT. (**C**) Resting time in the OFT. (**D**) Feeding latency in the NSFT. (**E**) Stationary time in the FST. (**F**) Number of correct responses for the 5-CSRTT. (**G**) Number of incorrect responses for the 5-CSRTT. (**H**) Accuracy rate of the 5-CSRTT. (**I**) Numbers of omissions for the 5-CSRTT. (**J**) Number of correct responses for the 5-CSRTT. (**K**) Number of incorrect responses for the 5-CSRTT. (**L**) Accuracy rate for the 5-CSRTT. (**M**) Numbers of omissions for the 5-CSRTT. Data analysis via one-way ANOVA and *t* test revealed that * *p* < 0.05, ** *p* < 0.01, and *** *p* < 0.001. Dunnett’s multiple comparisons test was performed for comparisons among groups. The data are presented in the form of mean values, n = 7.

Research shows that exposure to exogenous CORT can cause depression/anxiety-like behaviors. Current experimental results revealed that in the OFT, compared with those in the control group, rats in the CORT model group exhibited an obvious reduction in the distance traveled in the central region of the field, as well as an obvious increase in the OFT resting time (*p*_(B)_ < 0.001, *p*_(C)_ < 0.001, Figure 3B,C). In the NSFT, the food ingestion latency of the CORT model group was longer (*p*_(D)_ < 0.001, Figure 3D). In the FST, the immobility time of the CORT model group was longer than those in the control group (*p*_(E)_ = 0.0070, Figure 3E). These findings indicate that exposure to exogenous CORT can cause depression/anxiety-like symptoms in rats. In the subsequent 5-CSRTT, compared to rats in the control group, the rats under the exogenous CORT-exposed state had fewer correct responses (*p* = 0.0248, Figure 3F), an increased number of incorrect responses (*p* = 0.0393, Figure 3G), and a lower accuracy rate (*p* = 0.0284, Figure 3H), suggesting that the attention of the rats significantly decreased under a state of exogenous CORT exposure.

Next, the experimental animals in the exogenous CORT-exposed state with decreased attention levels were administered 3 mg/kg SA-4503 or 2.5 mg/kg or 5 mg/kg YL-0919 for 6 days, and the 5-CSRTT was subsequently performed. The results of the test revealed that the attention of the rats in the administration groups was substantially higher than those in the CORT model group (*F*_(4,31)_ = 4.501, *p* = 0.0055, Figure 3J; *F*_(4,31)_ = 4.585, *p* = 0.0050, Figure 3K; *F*_(4,31)_ = 4.850, *p* = 0.0037, Figure 3L). In contrast to the CORT model group, the number of incorrect responses of the rats in the 2.5 mg/kg YL-0919 administration group were lower (*p* = 0.0053, Figure 3K), and the accuracy rate was higher (*p* = 0.0060, Figure 3L). However, the number of correct responses in the 3 mg/kg SA-4503 and 5 mg/kg YL-0919 administration groups was higher (*p*_(SA)_ = 0.0020, *p*_(YL)_ = 0.0073, Figure 3J), the number of incorrect responses was lower (*p*_(SA)_ = 0.0022, *p*_(YL)_ = 0.0262, Figure 3K), and the accuracy rate was greater (*p*_(SA)_ = 0.0014, *p*_(YL)_ = 0.0167, Figure 3L). These findings suggest that exposure to CORT causes depression/anxiety-like behaviors and attention deficit in the rats. YL-0919 as a sigma-1 receptor agonist can significantly increase the attention of rats under an exogenous CORT-exposed state, which was consistent with the results obtained in rats under a physiological state, especially for the effect of 5 mg/kg YL-0919.

### 2.4. YL-0919 Improves Attention by Increasing the Expression of BDNF and Synaptic-Related Proteins and Dendritic Complexity in the mPFC of Rats Under a CORT-Exposed State

**Figure 4:**
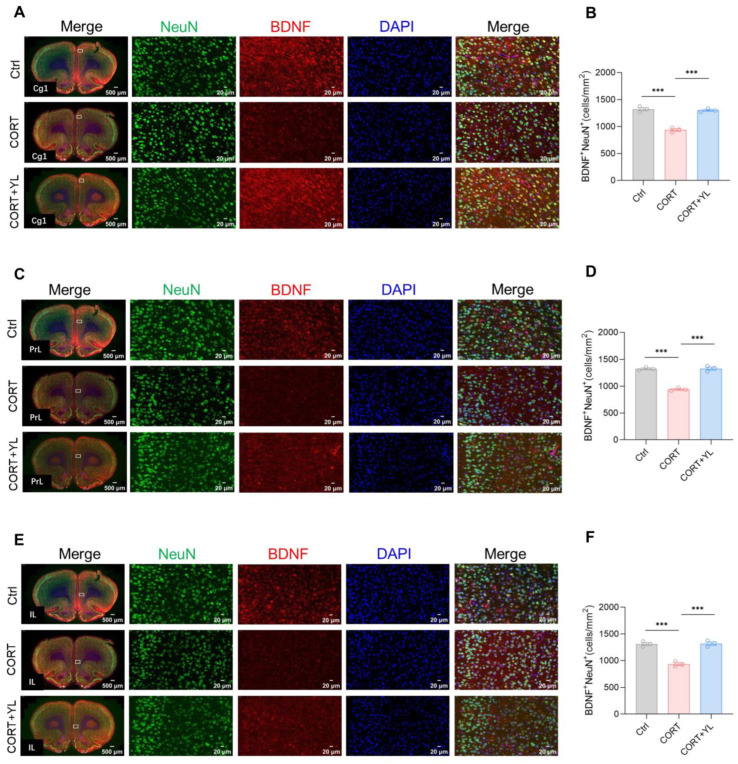
YL-0919 increased the number of mature neurons that secrete BDNF in the mPFC. (**A**) Representative images of the Cg1 for NeuN (green) and BDNF (red) immunostaining in the control, CORT, and CORT + YL groups. (**B**) Quantification of NeuN^+^ and BDNF^+^ double-positive cells in the Cg1 region. (**C**) Representative images of NeuN (green) and BDNF (red) immunostaining in the PrL in the control, CORT, and CORT + YL groups. (**D**) Quantification of NeuN^+^ and BDNF^+^ double-positive cells in the PrL. (**E**) Representative images of NeuN (green) and BDNF (red) immunostaining in the IL of the Ctrl, CORT, and the CORT + YL groups. (**F**) Quantification of NeuN^+^ and BDNF^+^ double-positive cells in the IL. Data analysis employed one-way ANOVA. Dunnett’s multiple comparisons test was performed for comparisons among groups. The data are presented in the form of mean values. *** *p* < 0.001, n = 3.

At the end of the behavioral experiment, we perfused and harvested the brains of three rats each from the control group, the CORT group, and the 5 mg/kg YL-0919 group. Immunofluorescence staining was used to visualize neurons that were double positive for BDNF and NeuN in the mPFC. The number of double-positive neurons represented mature neurons that secrete BDNF. The results revealed that, compared to the control group, exposure to CORT could reduce and YL-0919 could increase the number of mature neurons that secrete BDNF in the Cg1 (*F*_(2,6)_ = 75.38, *p* < 0.0001, Figure 4A,B), PrL (*F*_(2,6)_ = 109.4, *p* < 0.0001, Figure 4C,D), and IL (*F*_(2,6)_ = 58.47, *p* = 0.0001, Figure 4E,F) regions of the mPFC in rats under an exogenous CORT-exposed state, indicating that YL-0919 can increase BDNF from mature neurons.

**Figure 5:**
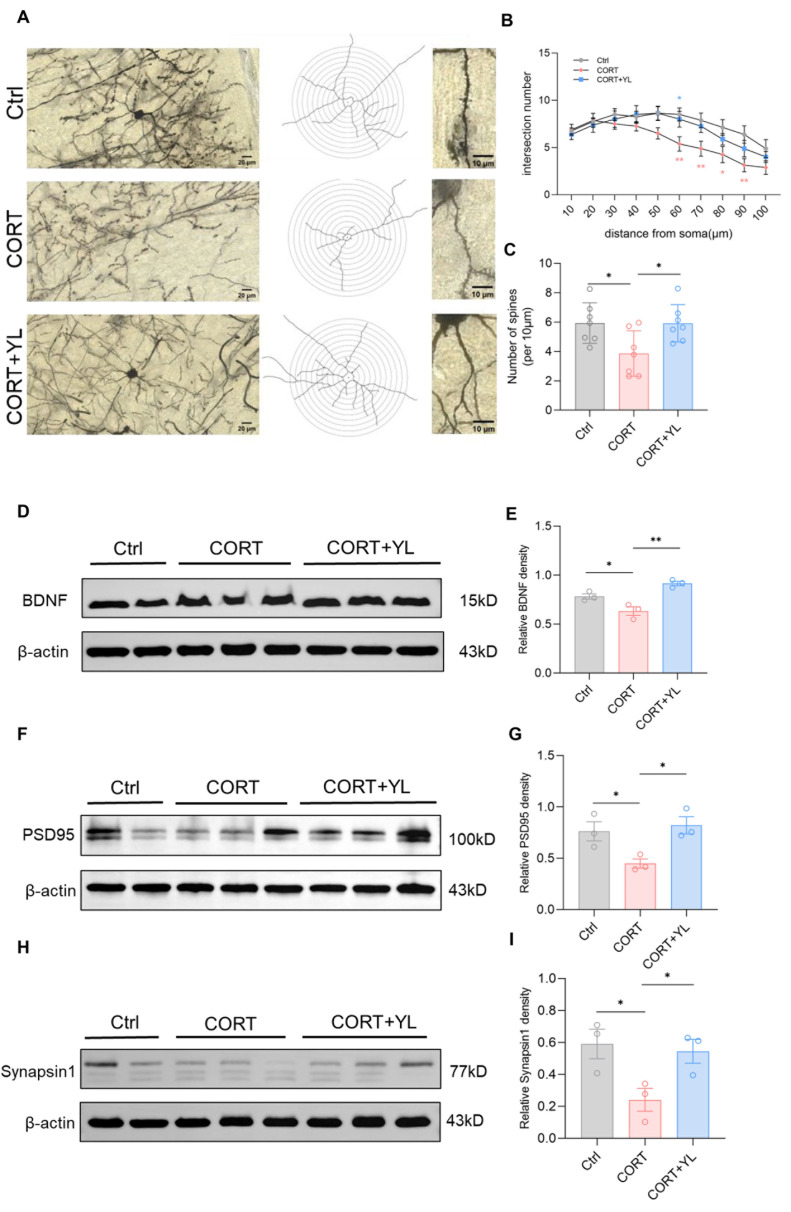
YL-0919 promotes dendritic plasticity and improves the expression of BDNF, PSD95, and synapsin1 in the mPFC. (**A**) Representative images of Golgi staining in the mPFC of the control, CORT, and CORT + YL groups. (**B**) Numbers of dendrite intersections in 10–100 μm concentric circles. (**C**) Number of dendritic spines per unit length. (**D**) Western blotting results showing the BDNF levels in the control, CORT, and CORT + YL groups. (**E**) Relative grayscale value of BDNF. (**F**) Western blotting results showing the PSD95 levels in the control, CORT, and CORT + YL groups. (**G**) Relative grayscale value of PSD95. (**H**) Western blotting results showing the synapsin1 levels in the control, CORT, and CORT + YL groups. (**I**) Relative grayscale value of synapsin1. In the western blotting, the spliced fragments originate from the same original image. Data analysis via one-way ANOVA and two-way ANOVAs revealed that * *p* < 0.05 and ** *p* < 0.01. Tukey’s multiple comparisons test and Dunnett’s multiple comparisons test were performed for comparisons among groups. The data are presented in the form of mean values, n = 3.

Brain tissues were collected from another three rats. The expression of BDNF (*F*_(2,6)_ = 18.88, *p*_(E)_ = 0.0026, Figure 5D,E), PSD95 (*F*_(2,6)_ = 6.819, *p* _(G)_ = 0.0285, Figure 5F,G) and synapsin1 (*F*_(2,6)_ = 5.552, *p*_(I)_ = 0.0432, Figure 5H,I) in the PFC was detected using western blotting. Compared with those in the model group, the protein levels of the rats in the 5 mg/kg YL-0919 group were higher, verifying that YL-0919 can increase attention by promoting the expression of BDNF and synaptic-related proteins. In addition, after the behavioral experiment, we harvested the mPFC from three rats each in the control group, the CORT model group, and the 5 mg/kg YL-0919 group for Golgi–Cox staining (Figure 5A). As depicted in the figure, the number of dendritic spines in neurons in the mPFC was reduced in the CORT group compared to the control group (Figure 5B); moreover, at the same distance from the cell body, the number of intersections of concentric circles was reduced (*F*_(2,18)_ = 5.042, *p* = 0.0183, Figure 5C). In addition, the number of dendritic spines in neurons in the mPFC was increased in the 5 mg/kg YL-0919 group compared to the CORT model group (Figure 5B). At the same distance from the cell body, the number of intersections of concentric circles increased (*F*_(2,18)_ = 5.042, *p* = 0.0183, Figure 5C), indicating that YL-0919 may increase attention by improving the expression levels of BDNF and synaptic-related proteins and promoting dendritic complexity in the mPFC.

## 3. Discussion

The 5-CSRTT is a classic behavioral protocol that mimics the evaluation paradigm of the human sustained attention continuous performance test (CPT) to test the attention behavior of rodents [28]. In this study, after the intragastric administration of 5 mg/kg YL-0919 to SD rats for 6 days, YL-0919 significantly increased the attention of SD rats under a physiological state and significantly alleviate attention deficit in SD rats under an exogenous CORT-exposed state. In addition, immunofluorescence staining and western blotting revealed that the expression level of BDNF, PSD-95, and synapsin1 in the mPFC of the YL-0919 groups increased. Golgi–Cox staining revealed that the number of distal dendrites in the CORT-exposed group decreased, and this change was abrogated after the intragastric administration of YL-0919. These results suggest that YL-0919 may improve attention by increasing BDNF expression and dendritic complexity in the mPFC.

Administering CORT repeatedly can lead to consistent behavioral and neurobiological changes. These changes mirror numerous core symptoms and neurobiological alterations linked to human depression [29], which is accompanied by attention deficit at the same time. In this study, we found that exogenous CORT exposure (200 μg/mL, drinking water for 21 days) caused depression/anxiety-like behaviors and attention deficit in the rats. These symptoms were reversed by intragastric administration of YL-0919 for 6 days. The results reveal that YL-0919 has a faster onset effect of enhancing attention, and the dosage is consistent with the dosage for the faster onset antidepressant effect [14]. The findings suggest that atomoxetine has undergone comprehensive research and demonstrated notable effectiveness continuously for 6 weeks in the treatment of adult attention deficit [30]. The malfunction of the noradrenaline transporter has been associated with a variety of neuropsychiatric disorders, such as major depressive disorder and attention deficit hyperactivity disorder [31]. Tricyclic antidepressants (TCAs) are occasionally employed as a second-tier treatment option for alleviating the symptoms of attention deficit in children and adolescents with this condition [32]. In terms of time, YL-0919 takes effect in attention deficit for 6 days, representing a faster onset effect. The research discovered that using methylphenidate (MPH) alone or in combination with citalopram led to an improvement in depressive symptoms [33]. Bupropion has been used to treat depression, attention deficit hyperactivity disorder and smoking cessation [12]. These studies indicate that some antidepressant drugs have the effect of improving attention deficit, and some drugs used for treating attention deficit can also be applied to improve depression. As a selective sigma-1 receptor agonist, YL-0919 can improve attention in SD rats, which provides a potential novel target for the faster drug therapy of attention deficit.

It is generally believed that BDNF and synaptic-related proteins can promote synaptic plasticity in the brain [34,35]. We think this may be one of the mechanisms by which YL-0919 enhances attention. In this study, we focused on changes in the expression of BDNF, PSD95, and synapsin1 in the mPFC. We found that YL-0919 administration obviously increased the expression of BDNF, PSD95, and synapsin1 and improved the neuronal dendritic complexity and the dendritic spine density in the mPFC. BDNF mainly plays its role through several signal transduction pathways [36] that affect synaptic plasticity, neurotransmitter release, and neuronal survival and regulate cognitive function. SG Kernie et al. reported [37] that BDNF expression was reduced in heterozygous BDNF knockout mice [BDNF (+/−)]. Compared with wild-type mice, BDNF heterozygous mice exhibited obesity and hyperactivity. These findings suggest that BDNF may be related to the pathogenesis of attention deficit. Moreover, a study using spontaneously hypertensive rats (SHRs) and a normal Wistar Kyoto rats as controls [38] revealed that BDNF levels in the hippocampi of SHRs were decreased and that treadmill exercise and MPH administration attenuated attention deficits and increased BDNF expression in the brain, suggesting that BDNF may be related to the mechanisms of attention deficit [36]. In addition, PSD-95 associates with relevant proteins as well as upstream and downstream signaling molecules, integrates excitatory signals, and activates downstream signal transduction pathways to regulate the synaptic state and maintain the synaptic structure [39]. Hye-Ji Kim et al. explore the cellular mechanisms underlying cortisol-induced cognitive impairment in rat pups whose mothers received repeated injections of CORT during pregnancy and reported that the expression of the NR2B subunit in the hippocampus of the rat pups was relatively high and the expression levels of PSD-95 and BDNF were relatively low [40]. Synapsin1 not only affects the structure of neuronal synapses but also regulates the secretion of neurotransmitters such as glutamate and acetylcholine through its phosphorylation. Synapsin1 can be used as a specific marker of synaptic vesicles, and its content indirectly reflects the number, density, and transmission performance of synapses [41]. When the sigma-1 receptor is activated, CaMKII is transported to the cell membrane and interacts with N-methyl-D-aspartate (NMDA) in pyramidal cells to rapidly activate CaMKII in the cell to phosphorylate intermediates of the ERK1/2 and mTOR pathways, thereby inducing the rapid synthesis of PSD95 and BDNF [42]. Moreover, the activated sigma-1 receptor combines with and modulates the ER stress response via the ER stress sensor IRE1. Meanwhile, it facilitates the expression of BDNF through the IRE1–xbp1 signaling pathway [43]. These studies all suggest that activation of the sigma-1 receptor can increase the expression of BDNF, PSD95, and synapsin1 in cells, thereby altering the dendritic complexity of neurons to improve attention.

Irregularities in the structure and operation of the PFC are generally considered to be associated with cognitive deficits in children, including deficits in working memory, inhibitory control, alertness, visual search, and focus [44]. The study revealed that during flexible goal-directed behavior, the PFC coordinates a wide range of goal-related information from the nervous system and prioritizes relevant information. This interregional coordination can be realized through the synchronization of separated high-frequency activation states and low-frequency normal excitation changes [45]. That is, the PFC is greatly involved in selective attention and attention shifting. Research findings have indicated that there are changes in the PFC circuit that cause weak PFC activation among attention deficit patients when trying to regulate attention and behavior [46]. Moreover, the PFC aids in focusing on important things, suppresses internal and external distractions, and appropriately divides and transfers our attention to multiple tasks [47]. Children with attention deficit presented significant abnormalities in the connectivity of the PFC and the striatum, cerebellum, and parietal lobe regions, whereas children with conduct disorder presented changes in the paralimbic system, including the ventromedial and lateral orbitofrontal and superior temporal cortex and specific border regions [48]. The PFC can direct the output of behavior through its complex projections to the motor and premotor cortex, to basal ganglia structures (such as the caudate nucleus and subthalamic nucleus), and through the pons to the cerebellum [49]. Therefore, lesions in other brain regions may also be part of the circuits of the brain regions involved in attention deficit. Many studies have shown that in adults and children with attention deficit, the gray matter volume of cortical and subcortical structures is reduced, and this reduction normalizes with age and stimulant drug treatment [50]. Clara S Li et al. revealed that the gray matter volume of the basal ganglia in children with attention deficit features is decreased, with the decrease in total volume being more significant in boys than in girls, and the role of the decrease in caudate nucleus gray matter volume in working memory dysfunction was elucidated [51]. In addition, researchers have reported that the PFC regulates the activity of the sensory thalamus through a basal ganglia pathway to filter sensory stimuli and regulate the function of attention. This study found that compared with those in the control group, the numbers of c-Fos and NeuN double-positive neurons in the mPFC, thalamus, and basal ganglia in the drug treatment group significantly increased. The research suggests that attention is an advanced cognitive function and multiple neural circuits in brain regions are involved in its regulation.

In conclusion, the persuasive evidence put forward in this study robustly indicates that YL-0919 can improve attention under a physiological state and exogenous CORT-exposed state. We used a model induced by exposure to exogenous CORT and revealed that as a selective sigma-1 receptor agonist, YL-0919 can increase the expression of BDNF and synaptic-related proteins and dendritic complexity in the mPFC by activating the sigma-1 receptor, thereby exerting an attention-enhancing effect. This study also suggests the sigma-1 receptor may be a potential novel target for the drug therapy of attention deficit in addition to the dopaminergic system.

## 4. Materials and Methods

### 4.1. Animals

A total of 60 male SD rats, specific pathogen-free (SPF) grade, weighing 270–290 g, were purchased from Si Pei Fu (Beijing) Biotechnology Co., Ltd. (animal production license: SCXK (Beijing, China) 2024–0001). During the experiment, the rats were kept in groups of four per cage and were limited to the amount of food according to the experimental requirements. Except for the period of exposure to exogenous CORT, drinking water was not restricted. The temperature of the housing environment ranged from 20 to 24 °C, and the humidity ranged from 50% to 60%. The mice were kept under a 12 h/12 h light/dark cycle (light on 8:00–20:00). All animal experimental procedures were approved by the Institutional Animal Care and Use Committee of the Beijing Institute of Basic Medical Sciences.

### 4.2. Pharmacology Administration

YL-0919 (white powder, purity ≥ 99.8%, #D5222-18-001) was purchased from Zhejiang Huahai Pharmaceutical Co. (Zhejiang, China). SA4503 was obtained from MedChemexpress. Both YL-0919 (2.5 mg/kg, 5 mg/kg) and SA4503 (3 mg/kg) were dissolved in physiological saline [52]. The rats received drug administration through intragastric gavage (i.g.). The dosage volume was set at 10 mL/kg, and the administration was carried out at the same time each day. This process continued until the behavior experiments were conducted.

On the basis of the average daily water consumption of 30–50 mL by the rats, a 200 μg/mL CORT solution was used to establish the CORT water drinking model [25]. Dissolve 200 mg of CORT (MCE, purity ≥ 99.76%, HY-B1618) powder in 10 mL of anhydrous ethanol. Use a 2 L clean beaker. Add the solution to 1 L of double-distilled water. Place it on a magnetic stirrer to stir evenly to ensure full dissolution of CORT. Store the excess CORT solution in a 4 °C refrigerator for future use.

### 4.3. 5-Choice Serial Reaction Time Task

The 5-CSRTT has four indicators for evaluating attention, namely, the number of correct responses, the number of incorrect responses, the number of omissions, and the accuracy rate. First, rats need to be trained to respond to an unpredictable visual signal randomly presented in one of five locations. The effects of drugs or experimental manipulation on the attention of animals can be demonstrated. According to the experimental requirements, the 5-CSRTT was divided into a training stage and a testing stage. The rats were fasted for 16–18 h before each training or test. Training stage: When the accuracy rate of the rats’ behavioral indicators (numbers of correct response/(numbers of correct response + number of errors) × 100%) reached 80%, the training of the next experimental condition was performed the next day. Dosing and testing stage: After the unified baseline required by the training was reached, the dosing was performed according to the experimental design, and the formal experiment began. During the whole process, each rat was trained and tested in the same experimental box.

### 4.4. Open Field Test

The rats were placed in the behavioral laboratory to adapt to the environment 1 h before the start of the open field experiment. During the experiment, the test rats were placed in the experimental box (100 × 100 × 40 cm, opaque) facing the wall of the experimental box. The rats were able to move freely in the experimental box for 5 min, and the distance travel and immobilization time of the rats in the central area of the experimental box were recorded. Before the start of the next round of experiments, the excrement of the rats in the experimental box was thoroughly cleaned using paper towels with 75% ethanol. The next round of tests was performed after the ethanol had completely volatilized to eliminate the influence of odor on the next test rat. SMART3.0 software was utilized for the purpose of recording and analyzing the behavioral patterns of the rats.

### 4.5. Novelty Suspended Feeding Test

The bedding was changed one day in advance, and the rats were deprived of food for 18–24 h (the water intake was maintained during this period). Before the start of the experiment, the test rats were placed in a new quiet environment (different from the rat breeding environment and other test environments). Clean bedding material with a thickness of approximately 1 cm was spread in the test box, and five grains of rat chow with a length of 5 cm were placed in the middle area and arranged in a cross shape. During the test, the SD rats were placed into the corner of the box with their back facing the food direction. The latency time of the rats for the first feeding behavior within 5 min was recorded. If the rat still did not eat at the end of the timing, it was considered 5 min.

### 4.6. Forced Swim Test

The test rats were placed in the behavioral laboratory to adapt to the environment 1 h before the start of the FST. The rats were placed in a transparent cylindrical glass cylinder (20 cm in diameter, 50 cm in height) filled with water to a depth of 30 cm, and the water temperature was set to 23 ± 1 °C by adjusting the ratio of cold to hot water. The night before the formal experiment, the rats were placed in a glass cylinder for 10 min before the experiment. After the start of the formal experiment, each rat was forced to swim for 6 min. A video of the rats swimming was recorded. While swimming, if the rat’s head was able to emerge from the water while the hind limbs were moving, but no other escape behavior was apparent, the rats were regarded as immobile. The total immobility time of the rats during the last 4 min of the FST was subsequently measured. The glass cylinders were blocked by black opaque plastic plates to avoid the mutual influence of experimental animals.

### 4.7. Western Blotting

After the behavioral experiments, the rats were sacrificed, and the brains were removed. The PFC was isolated and stored at −80 °C. The PFC tissues were ultrasonically ground 2–3 times, incubated at 4 °C for 30 min, and centrifuged at 12,000× *g* for 10 min, after which the supernatant was collected. The protein content of the tissues was quantified using the BCA method. Samples containing 40 μg of protein were separated using electrophoresis, transferred to PVDF membranes, and blocked with 5% skim milk. Anti-BDNF (1:1000, Servicebio, Wuhan, Hubei Province, China, GB11559), anti-PSD95 (1:1000, Servicebio, Wuhan, Hubei Province, China, GB11277), anti-synapsin1 (1:1000, Abcam, Cambridge, MA, USA, ab254349), and anti-β-actin (1:5000, Servicebio, Wuhan, Hubei Province, China, GB15003) were added to the samples and were incubated at 4 °C overnight. The membrane was washed with TBST for 5 min for a total of three times. HRP-conjugated goat anti-rabbit IgG (1:5000, Servicebio, Wuhan, Hubei Province, China, GB23303) was added to the samples, which were incubated at room temperature for 2 h in the dark. The images were developed using an Odyssey dual-color infrared fluorescence imager, and the integrated absorbance values of the protein bands were analyzed using ImageJ 1.54g software. β-Actin was used as an internal control, and the ratio of the integrated absorbance of the target protein to that of the internal control protein reflected the relative expression level of the target protein.

### 4.8. Immunofluorescence Staining

Upon completion of the behavioral experiments, the rats were anesthetized using pentobarbital sodium. Subsequently, the brains were harvested following perfusion with normal saline and 4% paraformaldehyde (PFA). The brain was fixed in 4% PFA overnight and stored at 4 °C. The fixed brain tissues were embedded in paraffin and sectioned. The sections were successively immersed in eco-friendly dewaxing solution I for a duration of 10 min, eco-friendly dewaxing solution II for 10 min, and eco-friendly dewaxing solution III for 10 min. Subsequently, they were placed in absolute ethanol I for 5 min, absolute ethanol II for 5 min, and absolute ethanol III for 5 min, and finally transferred into distilled water. Antigen retrieval was performed. After the antigen retrieval was complete, the sections were allowed to cool to room temperature naturally. The sections were placed in PBS (pH 7.4) and washed three times on a destaining shaker, each for 5 min. After the sections were shaken dry, a circle was drawn around the region of interest with a histochemistry pen, and BSA was added dropwise and incubated for 30 min. Anti-c-Fos (1:5000, Servicebio, Wuhan, Hubei Province, China, GB12069), anti-BDNF (1:300, Servicebio, Wuhan, Hubei Province, China, GB11559), and anti-NeuN (1:300, Servicebio, Wuhan, Hubei Province, China, GB11138) were prepared and added dropwise. The sections were placed flat in an incubator and incubated at 4 °C overnight. After that, the slides were placed in PBS (pH 7.4) and washed three times on a destaining shaker, each for 5 min. The corresponding secondary antibodies (HRP-labeled goat anti-mouse IgG 1:500, Wuhan, Hubei Province, China, GB23301; CY3-labeled goat anti-rabbit IgG 1:300, Wuhan, Hubei Province, China, GB21303; and Alexa Fluor 488-labeled goat anti-mouse IgG 1:300, Wuhan, Hubei Province, China, GB25301) were added to the sections, which were incubated at room temperature in the dark for 50 min. The slides were placed in PBS (pH 7.4) and washed three times on a destaining shaker, each for 5 min. DAPI staining solution was added on the slides, which were incubated at room temperature for 10 min in the dark. The slides were placed in PBS (pH 7.4) and washed three times on a destaining shaker, each for 5 min. Autofluorescence quencher B solution was added for 5 min, and the samples were rinsed with running water for 10 min. The coverslips were mounted with anti-fluorescence quenching mounting medium. After the slides were mounted, images were collected to obtain c-Fos^+^ and NeuN^+^ double-labeled sections and BDNF^+^ and NeuN^+^ double-labeled sections. The images were captured using CaseViewer 2.4 software and analyzed with ImageJ 1.54g software.

### 4.9. Golgi–Cox Staining

After behavioral testing, the rats were anesthetized with pentobarbital sodium, and the brains were collected after perfusion with normal saline. The brain tissue was quickly washed with normal saline to remove blood, placed in a G1069-30 mL bottle of Golgi staining and fixation solution, and stored at room temperature. The staining and fixation were replaced with Golgi staining solution to immerse the brain tissues completely, and the samples were placed in a cool and ventilated place at 26 °C and protected from light for 14 days. After the staining was completed, the tissue processing medium was changed for 1 h, and then the tissue processing medium was changed once every 3 days at 4 °C in the dark. The brain tissues were affixed to the tray of a vibrating slicer with 502 superglues and immersed in tissue processing solution. The 60 μm thick sections were affixed to glass slides with the assistance of a brush. After the sections were removed, tissue treatment solution was added dropwise to the surface of the brain tissue sections, which were allowed to dry slightly before development. The sections were washed with ultrapure water. The Golgi developer was added dropwise, and sections were developed in for 30 min and then washed in water. Excess water on the slides was removed, and the slides were mounted with glycerol-gelatin. All the tissues were scanned in brightfield scanning mode using a slide scanner and Pannoramic 250 FLASH scanner software. CaseViewer 2.4 software was used to capture the pictures and perform Sholl analysis.

### 4.10. Statistical Analysis

All the data are presented as means ± SEM and analyzed with GraphPad Prism 9.5 (GraphPad Software Inc., San Diego, CA, USA). For comparisons between two groups, either unpaired or paired t-tests were utilized. In the case of comparing three groups of distinct animals, one-way ANOVA was applied. When appropriate, two-way ANOVAs were used to conduct comparisons across multiple groups under diverse testing conditions. Tukey’s multiple comparisons test or Dunnett’s multiple comparisons test was performed for comparisons among groups. For all tests, *p* < 0.05 was considered statistically significant.

## Data Availability

The data is contained within the article.
